# Management of Brain Cancer and Neurodegenerative Disorders with Polymer-Based Nanoparticles as a Biocompatible Platform

**DOI:** 10.3390/molecules28020841

**Published:** 2023-01-14

**Authors:** Mehdi Bazi Alahri, Alhawarin Jibril Ibrahim, Mahmood Barani, Hassan Arkaban, Seyedeh Malahat Shadman, Soodeh Salarpour, Payam Zarrintaj, Javad Jaberi, Abduladheem Turki Jalil

**Affiliations:** 1 Department of Clinical Psychology, Faculty of Medicine, Shahid Beheshti University of Medical Sciences, Tehran 1971653313, Iran; 2 Department of Chemistry, Faculty of Science, Al-Hussein Bin Talal University, Ma’an 71111, Jordan; 3 Medical Mycology and Bacteriology Research Center, Kerman University of Medical Sciences, Kerman 7616913555, Iran; 4 Department of Chemistry, University of Isfahan, Isfahan 8174673441, Iran; 5 Pharmaceutics Research Center, Institute of Neuropharmacology, Kerman University of Medical Sciences, Kerman 7616913555, Iran; 6 School of Chemical Engineering, Oklahoma State University, 420 Engineering North, Stillwater, OK 74078, USA; 7 Medical Laboratories Techniques Department, Al-Mustaqbal University College, Babylon, Hilla 51001, Iraq

**Keywords:** central nervous system, nanotechnology, blood–brain barrier, drug delivery systems, polymer-coated nanoparticles

## Abstract

The blood–brain barrier (BBB) serves as a protective barrier for the central nervous system (CNS) against drugs that enter the bloodstream. The BBB is a key clinical barrier in the treatment of CNS illnesses because it restricts drug entry into the brain. To bypass this barrier and release relevant drugs into the brain matrix, nanotechnology-based delivery systems have been developed. Given the unstable nature of NPs, an appropriate amount of a biocompatible polymer coating on NPs is thought to have a key role in reducing cellular cytotoxicity while also boosting stability. Human serum albumin (HSA), poly (lactic-co-glycolic acid) (PLGA), Polylactide (PLA), poly (alkyl cyanoacrylate) (PACA), gelatin, and chitosan are only a few of the significant polymers mentioned. In this review article, we categorized polymer-coated nanoparticles from basic to complex drug delivery systems and discussed their application as novel drug carriers to the brain.

## 1. Introduction

Technology and medicine have gone hand in hand for many years. Consistent advances in pharmaceuticals and the medical field have saved millions of lives and improved many others [1]. Neurodegenerative diseases (NDs) are dangerous diseases that are defined as progressive neuronal damage in some regions of the nervous system which affect cognitive function, motor activity, and mental damage and consequently become irrecoverable neuronal loss [2,3,4,5]. Nowadays, we witness longevity growth in developed countries due to improving hygiene and quality of life, which is closely associated with remarkable growth in the prevalence of neurodegenerative (ND) illnesses in the older population. Neuroinflammation, stress, mitochondrial dysfunction, and metabolic problems are also prevalent reasons for the pathogenesis of ND diseases. Hence, they will have adverse emotional, social, and financial burdens on the healthcare system in the future. Accordingly, efforts to find an effective theranostic strategy have been focused on continuing to treat ND disorders at an earlier stage, which would enable the prevention of cognitive impairment and irreversible neuronal damage [6,7].

There are essential interfaces between the central nervous system (CNS) and the enclosed cells and blood vessels, in which endothelial cells are connected by tight junctions (TJ) and adherens junctions (AJ) [8]. The main function of the blood–brain barrier (BBB) consists of protecting neuron cells and maintaining the brain’s internal environment. Therefore, BBB breakdown assists the entrance into the brain of neurotoxic blood-derived products, cells, and pathogens and is related to immune responses which lead to various neurodegenerative issues. The main reason for this problem in the body is that many physiological obstacles, especially BBB, hamper effective therapeutic substances from reaching the site of action [9].

Systemic chemotherapy (using the free drug) is currently the main strategy for the treatment of cancer. The systemic chemotherapy method has many drawbacks including fast blood clearance, poor bioavailability, and low tumor targeting. To overcome these drawbacks, a high drug dose should be administered to cause the required anti-tumor effect which, in turn, causes severe adverse side effects. Therefore, some superparticles and drug delivery systems have been developed to enhance the accumulation of the therapeutic as well as the diagnostic agents in the tumors, decrease their side effects, and improve their efficiency, stability, polarity, and bioavailability [10,11].

**Figure 1:**
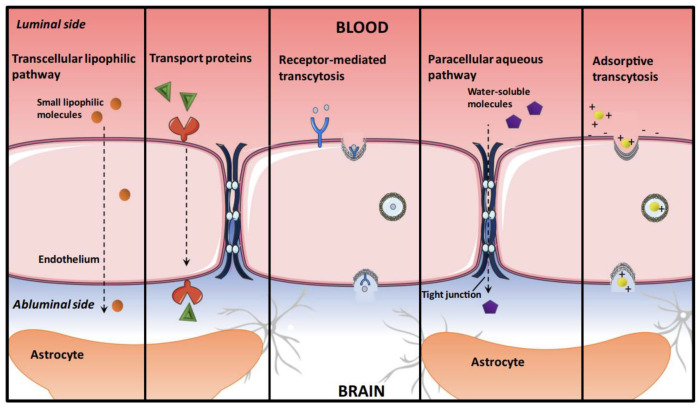
An overview of transport mechanisms across the blood–brain barrier (BBB), adapted from [43], Springer, 2020.

Nanotechnology research has found applications in many fields, from medicine and drug research to aeronautics and automobiles [12,13]. Active nanotech-based research has yielded several new advancements with wide-ranging applications [14,15]. New therapeutic efforts have been concentrated on the design of nanocarriers like polymeric nanoparticles [16,17], inorganic nanoparticles [18,19,20,21], green synthesized NPs [22,23,24], liposomes [25], nanofibers [26], hydrogels [27,28,29], Niosomes [30,31,32], and microemulsions [33,34] to transport therapeutic and diagnostic agents. In light of this fact, the above-mentioned nanoparticles (NPs) have attracted much attention thanks to their relatively high drug loading ability, lower or no systematic toxicity, controlled drug release, extended blood circulation time, capability of targeted delivery, and physical–chemical stability [35,36,37,38]. However, these properties for drug delivery rely on the type, size, surface chemistry, and polarity of the particles (Figure 1). On the other hand, molecule transformation is closely controlled by entering the brain parenchyma, then NPs penetrate the neuron cells by reaching the BBB, attending the surfactant in NP component and disrupting the TJ [39,40,41,42].

More importantly, the use of biopolymers in medical treatment has been regarded to make vigorous principles for peptides and proteins, as well as genes [44]. NPs coated in biopolymers in drug delivery permit them to simply penetrate the cells and release insoluble drugs to prevent their degradation. NPs can also be loaded with drugs by encapsulation within the polymer matrix adsorption, increasing the efficient loading and release of drugs in cells [45,46,47,48].

In this review, we try to focus on the problems of the BBB which appear in neurodegenerative diseases and how to design biocompatible polymer-coated and colloidally stable NPs to cure these disorders.

## 2. Brain Disorders

The World Health Organization (WHO) has recently declared that brain disorders are one of the primary morality causes in the world, mainly stemming from brain cancer or neurodegenerative diseases [49]. As most brain disorder diagnoses are highly invasive, time consuming, and expensive, it is imperative to develop non-invasive, cost-effective, and efficient tools for brain disorder characterization to reduce brain damage and improve longevity [50]. Surely, drug delivery in brain-based NPs coated in polymers opens a new avenue for solving these problems [51,52].

### 2.1. Brain Cancer

Tumors of the CNS are one of the challenges in medical care. They are typically located in the brain, which is a primary metastatic site for other organs’ tumors. The most prevalent types of brain tumors are gliomas. The different cell phenotypes of glia (astrocytes, oligodendrocytes, microglia, ependymal cells) can be the origin of gliomas. Based on the degree of cancer aggressiveness, gliomas are categorized from grade I to IV, in which the most malignant is IV [53]. Glioblastoma multiforme (GBM) is one of the most dangerous brain tumors in the world and kills many humans every year. The heterogeneity of the cellular and molecular properties of GBM complicates pharmacological interventions. GBM is a very invasive and recurring disease; furthermore, its clinical prognosis is poor, and finally, its mortality rates are high. Conventional therapeutic approaches like radiotherapy, chemotherapy, and surgical resection cannot be completely effective. The median overall survival time remains just 12–15 months after diagnosis, with less than 5% of people surviving longer than five years [54]. The complexity and delicacy of the structure and the primary function of the brain limit complete surgical resection. Due to the infiltrative growth behavior of GBM cells, the residual tumor cells can grow in the surrounding brain tissue. As the BBB protects these infiltrative cells from chemotherapy, resulting in high rates of cancer recurrence. Despite the conventional therapeutic regimen, alternative and targeted drug delivery are very essential to achieve better treatments for brain cancers [55].

### 2.2. Neurodegenerative Diseases

Neurodegenerative diseases are a group of neurological disorders in which the nervous function and anatomy in which both the peripheral and central nervous systems are gradually destroyed. These diseases are age-related; therefore, the number of cases is expected to rise as lifespans in many countries continue to increase. As the world grows older, unfortunately, the number of people with neurodegenerative diseases will increase. Furthermore, the importance of these diseases quickly becomes more serious than other diseases. They have relatively long durations and high treatment costs, so neurodegenerative diseases become a serious challenge for both patients and community healthcare. The etiology of these disorders is complex, but combinations of genetic factors and environmental conditions as contributing causes may play roles in neuronal dysfunctions. Alzheimer’s disease (AD) and Parkinson’s disease (PD) are two of the most common [56,57].

#### 2.2.1. Alzheimer’s Disease (AD)

Alzheimer’s disease (AD) is a neurological illness that induces progressive dementia. It is a progressive, long-term, and age-related neurodegenerative illness that manifests symptoms in three stages: severe, mild, and moderate dementia. Short-term memory loss is typical in AD, which is followed by cognitive decline, mental illnesses, behavioral abnormalities, and coordination problems, finally leading to death [7].

Many aspects of the neuropathogenesis of AD have not yet been completely elucidated due to the diversity of AD pathogenesis. Numerous clinical data suggested hypotheses concerning AD, which is a progressive accumulation of protein aggregation in the brain that causes neurodegeneration [58]. The Aß amyloid protein cascade hypothesis, oxidative stress theory, mitochondrial dysfunction, tau protein hyperphosphorylation theory, and the neuroinflammatory response are among the ideas hypothesized for the neuropathogenesis of AD. Other pathogenic pathways for AD have been identified as melatonin deficit, metal ion metabolism disturbance, cholinergic damage, immunological abnormalities, and so on [7,58,59]. The hypothesis of AD pathogenesis asserting that the impairment of cholinergic neurotransmitter systems may be the cause of the suppression of acetylcholine by Acetylcholinesterase (AChE) activity and the activation of the glutamatergic system has been considered to develop drugs based on these mechanisms [60].

#### 2.2.2. Parkinson’s Disease (PD)

Parkinson’s disease (PD) is the second most common progressive neurodegenerative disease. The pathophysiology of PD in human postmortem studies recognizes the neurodegeneration or death of neurons in locus ceruleus, the substantia nigra par compacta, and other neuronal populations. Based on the results of scientists’ research, the environment/lifestyle, and genetics (PARK-SNCA, PARK-PArkiN, etc.), several molecular events and hallmarks are considered the main causes of this disease. Mitochondrial dysfunction, α-synuclein misfolding, neuroinflammation, oxidative stress, aggregation, and impaired calcium homeostasis are involved in the pathogenesis of PD. Lewy bodies and Lewy neurites are intracellular disease-related protein aggregates and neuropathological hallmarks of PD. They are mostly present in pigmented neurons in the substantia nigra; however, they have been found in other central and peripheral neuronal populations [61,62].

The neuronal dysfunctions appearing in PD present with motor and non-motor symptoms. The main motor symptoms are tremors at rest, muscle rigidity, and bradykinesia, which can be related to the damage of dopaminergic neurons. On the other hand, the loss of non-dopaminergic neurons causes other motor symptoms relating to walking, balance, and posture. The quality of life in Parkinson’s patients is significantly reduced due to these various complications. Besides these motor symptoms, the non-motor symptoms affect the PD patients’ life, such as their mood conditions, sleep disorders, cognitive dysfunction, and dysautonomia [7].

## 3. The Challenge of the BBB

The application of drugs used for brain disorders is restricted, since they must overcome barriers. The BBB is a vasculature of the CNS separating the circulating blood from the brain’s extracellular fluid. The BBB is a semi-permeable and highly selective membrane barrier that imposes various obstacles to the transfer of many molecules. The BBB consists of a continuous layer of endothelial cells joined by tight junctions, transport proteins (efflux pumps), and extracellular and intracellular enzymes in the brain parenchyma which are called the physical barrier, transport barrier, and, enzymatic barrier, respectively [63]. This structure can control the transport of all substances and differentiate between molecules with specified components. Some essential substances such as glucose, amino acids, and hormones are transferred by transporters through the blood to the brain and vice versa. The influx of substances into the brain’s capillary endothelial cells and choroid plexus epithelial cells is facilitated by uptake transporters, while the materials are exported from the cells by efflux transporters. The efflux transporters on the blood-facing membrane of the BBB can be a threat to return drugs to blood circulation [7].

In transporting drugs to the brain, many factors influence the delivery and effectiveness of therapeutic agents through the BBB. For example, if drug molecules bind to non-transporters, they cannot be effective and pass through the BBB. Furthermore, the solubility and the molecular weight of each structure have an important role, since the lipophilic low-molecular-weight molecules that are not ionized at physiological pH can cross the BBB by diffusion. Moreover, other various factors could be effective for the delivery of drugs, e.g., the membrane or luminal surface of the brain capillary, the composition of cerebrospinal fluid (CSF) or interstitial fluid, and the functional groups or surface charge of therapeutic structures, so the prediction of drug transportation through the BBB may be very complicated [64].

According to the brain’s protective mechanisms and the importance of drug transportation to this organ, various strategies have been studied to overcome or bypass these obstacles. Therefore, maintaining normal body functions and reducing the post-delivery toxicity of the drugs is highly essential. Increasing the bioavailability and targetability of therapeutic substances in CNS-related diseases could be achieved by drug delivery systems, but some of them demonstrate invasive or nonspecific pathways [7,64].

## 4. Coating Polymers

Many types of nanocarriers have been developed for treating brain disorders [65]. Polymer-based therapeutic agents have been explored for the treatment of neurodegenerative diseases due to various fascinating advantages of polymers such as great biocompatibility, nontoxicity, controllable degradation rate, tunable architectures, the possibility of multiple interactions between amyloidogenic protein/peptide and polymer, and excellent in vivo stability [66].

In this section, we will look at some of the most commonly used coating polymers for neurodegenerative disorders.

### 4.1. Polysorbate (PS)

PSs are nonionic synthetic surfactants. This group of surfactants with uncharged head groups is an important subgroup of surfactants made up of poly ethoxy Sorbitan fatty acid esters. PSs have long been used in pharmaceuticals and food additives like emulsifiers and stabilizers [67,68].

**Figure 2:**
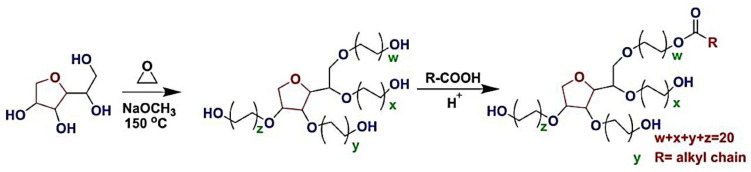
PS synthesis of Sorbitan, adapted from [76], MDPI, 2021.

**Figure 3:**
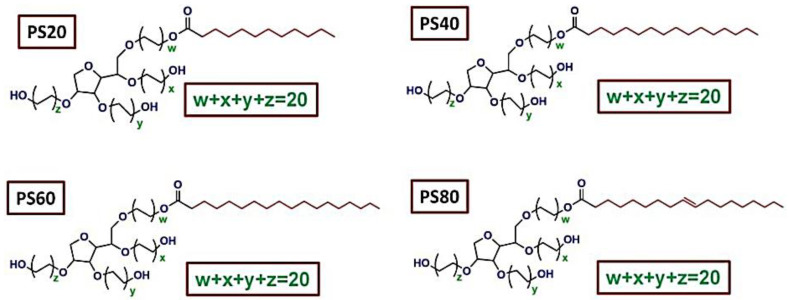
Structures of several PSs, adapted from [76], MDPI, 2021.

PSs have a Sorbitan ring with polyethylene oxide (PEO) attached to hydroxyl groups as their backbone structure. The four hydroxyl groups of the Sorbitan ring are conjugated to various numbers of ethylene oxide subunits. PSs are made chemically in two steps: Sorbitan is ethoxylated, then esterified with fatty acids. Figure 2 depicts the PS synthesis scheme, and Figure 3 shows the type of fatty acid ester connected with the PEO Sorbitan portion of the molecule, with the number following PS. PSs contain hydrophilic PEO segments and hydrophobic fatty acid segments. As a result, amphiphilic PSs can be used as effective surfactants [69,70,71]. PSs have been used as solubilizers and emulsifiers for hydrophobic pharmaceuticals, as well as protein stabilizing agents, and as drug carriers for both hydrophilic and hydrophobic medications [72,73,74,75]. PSs have also pulled in awesome consideration as brain-targeting coating materials that can encourage the transport of drug carriers over the blood–brain barrier (BBB). PSs have various points of interest, such as commercial plenitude and ease of chemical alteration, which render them reasonable for different biomedical applications. Hence, PSs have been broadly utilized as emulsifiers and stabilizers in different drug formulations and nourishment additives [76]. Additionally, PSs exhibit high biocompatibility and low toxicity and are thus advantageous for use as drug carriers. Owing to their amphiphilic nature, PSs have been used as solubilizers of hydrophobic drugs, proteins, and inorganic nanoparticles. Therefore, PS-based drug carriers can carry multiple drugs for combination therapy. Particularly, PS-drug conjugates and PS-coated drug carriers have great potential to treat central nervous system-related diseases because PS coatings can improve the crossing of the BBB [77].

PS 80 (Tween 80) has been used in the formulation of therapeutic monoclonal antibodies due to its unique properties. These properties include low toxicity, good protein stabilization, and biocompatibility. PSs have been used in vivo and in vitro to improve the permeability of various drugs. PS 80 is an emulsifier that facilitates drugs crossing through the BBB [78]. PS80 increases BBB crossing in epithelial BBB cells by adsorbing apolipoprotein onto nanoparticles, resulting in LDL receptor-mediated transcytosis [79,80]. PS 80-coated R-HCl loaded nanoparticles can also be an efficient drug delivery system in the treatment of Parkinson’s disease to revert the neurodegeneration with the strategy of coating the chitosan nanoparticles which enhance the brain targeting of the encapsulated drug [81].

### 4.2. Polyethylene Glycol (PEG)

PEGs are also known as Macrogols [82]. PEG has become well known due to its great structural flexibility, lack of steric hindrances, amphiphilicity, biocompatibility, and high hydration capacity and has lots on interest in drug delivery applications [83,84]. PEGs have extremely active functional terminals and are electrically neutral at all pH levels [85].

The FDA has approved PEG for intravenous, oral, and cutaneous use in humans [86, 87]. As a result, biocompatibility, circulation time, and aggregation are all improved [88]. PEG has been used to coat various polymeric nanoparticle systems. Surprisingly, the length of the PEG chain has an impact on the penetration of polymeric nanoparticles into the extracellular region of the brain. PEG-coated nanoparticles have recently grabbed great attention in the treatment of AD due to the vast biological efficiencies of PEG [17,89]. PEG is a polymer of choice in drug delivery systems and is popular due to its tunable properties and well-established safety profile. PEG coating of NPs can make a highly suitable strategy for improving efficiency in the systemic delivery of therapeutic agents and studying how the properties of PEG coatings influence NP biodistribution and the ability to penetrate the brain [16,90].

### 4.3. Chitosan

Chitosan is a linear polysaccharide, and because of its low cost, availability across a wide variety of molecular weights, and biodegradability, it is one of the most commonly used natural polymer nanoparticles for medication delivery [91]. It also has special biological features like anticancer, antibacterial, and antioxidant capabilities [92]. Chitin, a natural polymer derived from crustaceans or fungi, is partially N-deacetylated to synthesize chitosan [93]. Chitosan contains three different kinds of functional groups (amine, primary, and secondary hydroxyl) which could be used to make a variety of chemical modifications. The molecular weight, degree of deacetylation, and chemical modifications can all affect its biodegradability [92]. Chemical cross-linking, ionic gelation, and microfluidic synthesis are all ways that can be used to prepare chitosan nanoparticles [94,95]. These nanoparticles have shown great potential in brain drug delivery because of their positive charge, which increases cell uptake and makes them appropriate for loading with negatively charged therapeutics [96]. As an example, antibody-modified PEG–chitosan nanoparticles showed significant brain absorption, which was attributed to the antibody’s synergy with the positive chitosan charge [97]. Despite this, chitosan nanoparticles have drawbacks, including inadequate drug loading effectiveness on hydrophobic substrates and poor molecular weight control [98]. However, many advantages, including its ability to improve the bioavailability of drugs, efficiency in targeted drug delivery, reducing side effects, increasing drug effects at the target site, and increasing drug stability have attracted many researchers to the design of brain delivery systems in this way. Importantly, chitosan can open TJs with its ability to cross the BBB to provide sufficient chitosan-based DDSs for brain drug delivery applications and represent a better alternative to conventional drug formulations in brain diseases [99,100,101].

### 4.4. Poly-Ɛ-Caprolactone (PCL)

PCL is a biodegradable, FDA-approved polyester that has been utilized in a variety of applications, including sutures, implants, contraception devices, and drug delivery systems [102,103]. PCL is made up of repeated hexanoate units and can be broken down in the body by hydrolysis into 6-hydroxy caproic acid [104], which can subsequently be converted into adipate [105] and catalyzed to CO_2_ [106]. The ring-opening polymerization of 𝜖-caprolactone or the condensation polymerization of 6-hydroxyhexanoic acid is used to prepare it. Due to PCL’s insolubility in water, di-block PEG-b-PCL copolymers are commonly used to prepare PCL-based nanoparticles. Standard procedures such as film dehydration, microfluidics, emulsion, and solvent displacement can be used to prepare these nanoparticles [107]. Drug delivery for neurological diseases has also been examined using PCL-based nanoparticles [108]. For example, in an intracranial glioma tumor-bearing in vivo model, peptide-functionalized PEG–PCL micelles demonstrated significantly better transport ratios and increased accumulation in an in vitro BBB model [109]. PCL, on the other hand, has a low degradation rate, making it inappropriate for use as a drug delivery method [110]. Changing the molar mass or coating it with alternative polymers, such as PLA, might alleviate this problem [111]. Hence, it has been used as a suitable coating in the design of the brain delivery system [52,112,113].

### 4.5. Polyacrylic Acid (PAA)

**Figure 4:**
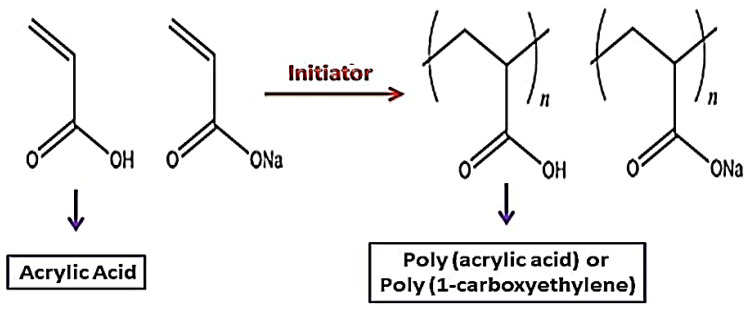
Synthesis and chemical structure of poly sodium acrylate (NaPAA) and PAA, adapted from [126], MDPI, 2020.

PAA, also known as poly 1-carboxyethylene, is a high-molecular-weight synthetic polymer produced from acrylic acid monomers. PAA is typically formed with the use of an initiator and free radical polymerization (Figure 4). Poly (1-carboxyethylene) is a low-cost polymer that has been commercialized [114,115,116].

PLA, a common pH-responsive polymer [117], has traditionally been used as a hydrophilic section in amphiphilic or amphipathic block copolymers with a wide range of properties [118]. PAA is an acrylic acid polymer containing a carboxylic group (–COOH) on each monomer unit end which is attached to the vinyl group (Figure 4). PAA, a thermoplastic polymer with many carboxyl groups, has a high bioavailability and can be used as a surface modification for biological nanomaterials [119]. PAA is a biocompatible, nontoxic, and biodegradable polymer that has attracted a lot of attention in recent years [120,121,122]. PAA nano-derivatives can be made by chemically modifying carboxyl groups, and they have better chemical characteristics than untreated PAA. PAA is used in a variety of industries, including adhesives, coatings, packaging, pharmacology, and other medical and biological fields [123]. PAA offers excellent medication storage and delivery capabilities due to its nontoxicity and absorption qualities. According to this, this polymer can be a suitable choice for the brain delivery system [124,125].

### 4.6. Poly (Lactic-co-Glycolic Acid) (PLGA)

PLGA is a type of linear copolymer that can be synthesized in a variety of ratios of the two monomers lactic acid and glycolic acid [127]. The FDA has approved PLGA for medical applications like drug delivery devices and biomaterials. The hydrolytic de-esterification of the PLGA copolymers is followed by the clearing of their monomeric anions, lactate, and glycolate, making them nontoxic and biodegradable [128,129]. The transformation of glycolic acid and lactic acid can alter the rate of degradation, degree of crystallinity, and mechanical strength, and therefore release kinetics and drug loading. Polylactic acid (PLA) is a crystalline hydrophobic polymer because of its methyl side chains, whereas polyglycolic acid (PGA) is a rigid and hydrophilic polymer with low mechanical strength [130]. As a result, PLGA copolymers with a higher PGA:PLA ratio are more hydrophobic, which means they degrade and release drugs at a slower rate [131]. PLGA can be prepared by using several methods: Segmer assembly polymerization [132], ring-opening polymerization [133], and the polycondensation process [131]. PLGA nanoparticles can be prepared from PLGA copolymers, utilizing processes like emulsion, nanoprecipitation, solvent co-evaporation, and spray-drying [134]. Soft lithography can also be used to synthesize non-spherical nanoparticles (e.g., cylindrical shapes) [135]. The terminal carboxylic acid groups can be used to introduce required surface modifications [136]. As a result, a wide range of pharmacological compounds, including anti-inflammatory medicines, antibiotics, chemotherapeutics, and proteins, have been integrated into PLGA nanoparticles [134]. For crossing the BBB, a variety of PLGA formulations have been investigated [137]. PLGA may be a well-known choice as a biodegradable medication carrier. The debasement rate of PLGA and the discharge of typified drugs can be controlled by the physicochemical properties of the polymer such as atomic weight, hydrophilicity, and lactide (LA) to glycolide (GA) proportion. So, PLGA-based nanoparticles have a higher effective half-life, bioavailability, and efficacy, and can be efficiently delivered to the brain by intranasal administration as well [138,139,140].

### 4.7. Hyaluronic Acid (HA)

**Figure 5:**
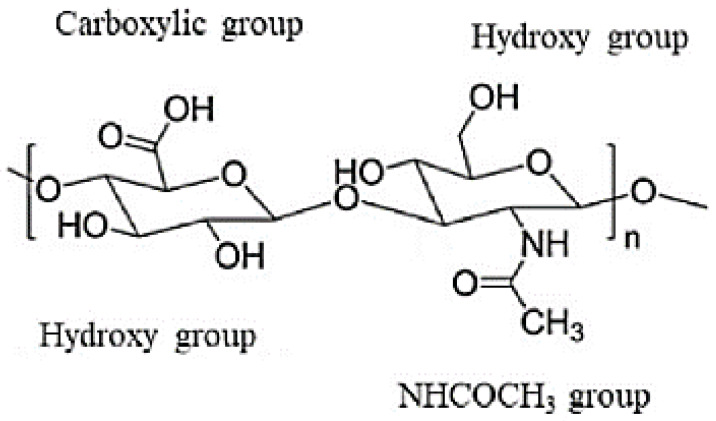
Chemical structure of hyaluronic acid [146].

Hyaluronic acid (HA), a biocompatible, biodegradable, and chemically adaptable molecule, has received more and more interest in the biomedical community during the past ten years. The extracellular matrix (ECM), which contains a significant amount of HA, is crucial for preserving cellular homeostasis and interaction [141,142]. Although HA is a naturally occurring substance with favorable cell interactions, low immunogenicity, mild antigenic characteristics, and the ability to be chemically modified, it has poor mechanical qualities, is expensive to produce, and is not very reproducible [143,144]. It has been demonstrated that HA is involved in tissue development, embryonic development, angiogenesis, cell migration, and proliferation. With polar and apolar moieties in the polymer structure, HA is naturally hydrophilic and allows for chemical interaction with a variety of chemical agents (Figure 5). Its molecular weight (MW), which in turn relies on the source, determines its structural, physicochemical, degradable, and biological features [145]. The HA-based polymer has been a terrific idea for the examination of tumors, especially for brain tumors, and is ideal for generating a more biocompatible system indicative of a healthy condition. In the CNS, HA plays a significant role in a variety of cell activities, including cell migration, proliferation, differentiation, and others. Medication thickening, the formation of conjugates when HA and its derivatives are utilized as carriers, sustained release, and improved drug targeting have all been demonstrated [146]. Therefore, this polymer may be a promising option for treating neurodegenerative diseases and brain tumors [147].

### 4.8. Cyclodextrins (CDs)

**Figure 6:**
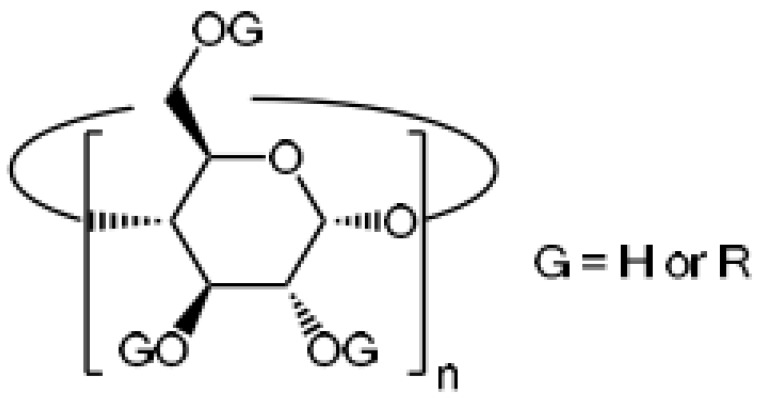
Chemical structure of cyclodextrins [148].

CDs are cyclic oligosaccharides made from starch by enzymatically cleaving the amylose helix. These ring-shaped molecules are very hydrophilic because their many hydroxyl moieties face outward. On the other hand, due to the glucosidic oxygen linkages, the inner side of the cavity is less hydrophilic. With the help of this structure, CDs can incorporate other less hydrophilic compounds (guests) into the cavity, creating what are known as host–guest inclusion complexes. Because of the CD cavity’s molecular size, the majority of medications, tastes, cosmetic compounds, insecticides, etc., can be molecularly enclosed there. The size of the guest affects the complex’s stoichiometry. Through particular synthetic processes, the many hydroxyl groups are easily changed to create different CD derivatives [148] (Figure 6).

The potential of cyclodextrins to actively remove lipids from cell membranes and to offer an appropriate carrier system for drug delivery has led to an ongoing increase in interest in CDs as therapeutic agents. The goal of creating new CD derivatives is to enhance CD bioavailability, biocompatibility, and therapeutic results. Due to their ability to reduce cell toxicity and hide the bitterness of many active compounds or adjuvants, CDs have the potential to improve medication solubility, bioavailability, and chemical stability. This has been linked to the fact that CDs’ physicochemical structure makes it possible for drug candidates to be trapped inside of their cavities, enabling the administration of hydrophilic or hydrophobic medications into bodily tissue. Many CD derivatives have been created to increase these molecules’ water solubility relative to their natural counterparts [149]. There are two key benefits to the CDs’ capacity to build inclusion complexes. In order to assist their aqueous dissolution, they first aid in solubilizing poorly hydrophobic substances by employing CD as a so-called carrier partner. The hydrophobic interior of the CDs makes it simple to encapsulate hydrophobic molecules of interest, while the hydrophilic outside of the CDs renders the entire complex water soluble. The second benefit of inclusion complex creation is that it significantly alters the properties of the target molecule (in our example, “drugs”). This includes altering the drug’s stability, bioavailability, oral absorption, and interactions with biological membranes and cells. CDs are widely used as drug delivery carriers through nasal mucosae and ocular, dermal, intestinal, and brain barriers because they improve the delivery and bioavailability of hydrophilic, hydrophobic, and lipophilic drugs. CDs have also been widely used to improve biocompatibility and bioavailability when combined with active drug compounds, thereby increasing drug efficacy [150].

### 4.9. Human Serum Albumin (HSA)

**Figure 7:**
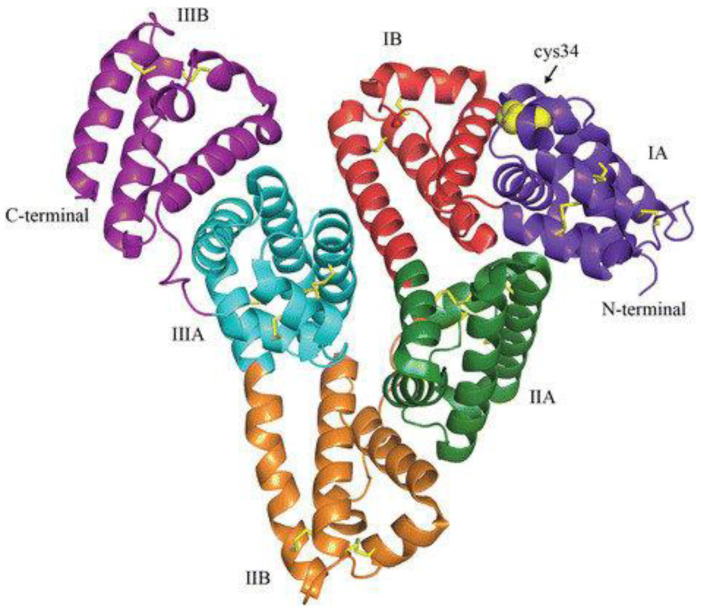
The 3D structure of HSA [153].

The most prevalent protein in plasma, HSA, is a monomeric multidomain molecule that serves as both the primary moderator of fluid flow across bodily compartments and the primary determinant of plasma oncotic pressure. HSA has a remarkable ability for complex formation, acting as a store and transporter for a variety of endogenous and foreign substances. Indeed, HSA serves as the primary carrier for fatty acids, influences the pharmacokinetics of many medications, allows for the metabolic modification of some ligands, neutralizes potential toxins, makes up the majority of the antioxidant capacity in human plasma, and exhibits (pseudo-)enzymatic properties [151]. HSA is an excellent choice for nanoparticle preparation because it has been demonstrated to be biodegradable, nontoxic, simple to purify, and soluble in water. This makes it easy to administer via injection. It has been demonstrated that adding the right medications to nanoparticles can prevent the pharmacologically active compounds from prematurely degrading or inactivating after injection as well as during storage [152]. 3D structure of HSA is shown in Figure 7.

## 5. Polymer-Coated Nanoparticles

Nanotechnology is helping to considerably improve, even revolutionize, many technologies and industry sectors. Nanomedicine is the medical application of nanotechnology. Nanomedicine ranges from the medical applications of nanomaterials and biological devices to nanoelectronic biosensors, and even possible future applications of molecular nanotechnology such as biological machines [154,155,156]. Nanoparticles are extensively used to increase the efficacy of a drug and reduce side effects through direct targeting and site-specific controlled delivery [157]. However, due to the toxicity of inorganic nanoparticles, polymers are generally used as nanoparticle carriers to reduce toxicity and increase cellular uptake. Table 1 summarizes some advantages and disadvantages of four common nanoparticles in brain delivery for neurodegenerative disorders.

Even though the already specified drugs are compelling within the treatment of neurodegenerative diseases (NDDs), the blood–brain barrier impedes their treatment and determination given its nearness to the central CNS illness [116,161,162,163]. Due to their low bioavailability and pharmacokinetic characteristics, traditional medicines, such as protein macromolecules, are limited in their therapeutic applicability. Subsequently, the consideration of NPs with neurotrophic impacts has become a modern hotspot within the NDDs study field [126]. Nanotechnology’s rapid growth and wide range of uses necessitate a strategy for creating safe NPs [164]. The architecture of the surface is a key role in the physicochemical properties of metallic particles that influences their mechanism of action in organic or natural environments [10].

Graphene is one of the prominent compounds that is used frequently in the therapy and diagnosis of neurodegenerative disorders. For the better function of graphene, the surface chemistry of graphene was modified with hydrocarbon, hydroxyl, aldehyde, and carboxyl groups and was studied by means of computational chemistry [165]. Paola Ginestra illustrated porous framework generation by electrospinning an arrangement containing PCL and graphene powder with completely different percentages. The effect of graphene on filament shape and mechanical properties in pliable testing was investigated, as well as the effect of electrospun substrate composition on neural stem cell separation. The researchers refined rat stem cells on fibrous platforms to study the effect of filament action on cell morphology and neuron divergence [166]. Rossana Rauti et al. demonstrated that slight graphene oxide nanosheets (s-GO) target specific presynaptic vesicle release. They propose that s-GO chips diminish the accessibility of the transmitter, using its advancing quick release and ensuing exhaustion, driving a decline in glutamatergic neurotransmission. After injecting s-GO into the hippocampus in vivo, ex vivo patch clamp recordings from brain slices revealed a substantial decrease in glutamatergic synaptic activity compared to saline infusions 48 h later [167].

**Figure 8:**
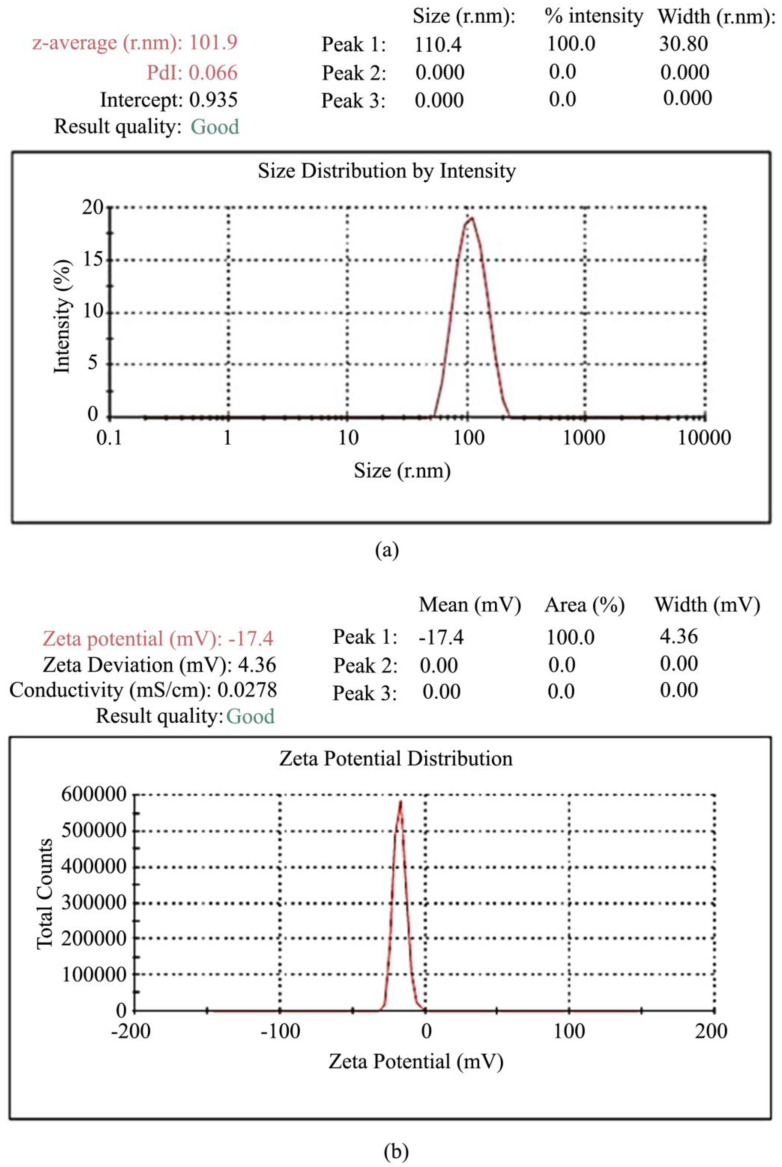
(**a**) Graph representing the particle size analysis and (**b**) Zeta potential of the optimized nanoparticles (CGA-NPs), adapted from [168], scientific research, 2020.

Chlorogenic acid (CGA) is a polyphenolic compound that has potential anti-inflammatory and antioxidative features and has risen as a promising compound in ameliorating NDDs. Owing to its destitute steadiness, bioavailability, and release kinetics, CGA required an appropriate nanocarrier-based pharmaceutical plan for targeting NDDs. Vinayak Agarwal et al. considered the in-silico approval of CGA as a successful therapeutic agent targeting different NDDs, through the creation of a polymeric nanoparticle-based carrier framework to overcome its pharmacological confinements and progress its stability. A fruitful in silico approval was conducted utilizing atomic docking strategies at the side synthesis of polymeric nanoparticles loaded with CGA (CGA-NPs) by ionic gelation strategy. The results displayed a mean size of 101.9 ± 1.5 nm with a polydispersibility of 0.065 and a ZP of −17.4 mV (Figure 8). The release kinetics information from in vitro also appeared to support the release of CGA from the NPs after taking the first-order kinetics, recommending the suitable planning of nanoformulation [168].

Igor M. Pongrac and colleagues evaluated the effect of different surface coatings on the cellular uptake and toxicity of Ag NPs against murine neural stem cells (mNSCs). Some silver NPs were prepared with different surface coatings including cetyltrimethylammonium bromide (CTAB), poly-L-lysine (PLL), poly(vinylpyrrolidone) (PVP), sodium bis (2-Ethylhexyl)-sulfosuccinate (AOT), and bovine serum egg whites (BSA). The findings demonstrated that Ag NPs stabilized with various surface coatings had various toxicities and uptake effects in MNCs. For all of the Ag NP types tested, macropinocytosis was determined to be the most widely used tool in mNSCs. These findings add to the body of knowledge necessary for assessing the security of new nanostructures [169].

Liu et al. prepared PS NPs and attached an iron chelator to them, demonstrating their ability to protect neurons in vitro. The developed NPs have the potential to apply as a transfection vector in Parkinson’s treatment, as well as an elective infection to reduce the immune response in blood circulation [170].

Trapani et al. used chitosan nanoparticles for the encapsulation of dopamine acid (DA) as a safe vector to pass through the blood–brain barrier. Furthermore, the sum of DA and chitosan NPs within the striatum is greater than that of DA alone [171]. In a separate study, Javier Garcia et al. reported the production of a family of polymeric NPs and the DA loaded within NPs by its reversible coordination complexation. The results revealed a loading efficiency of up to 60%. DA nanoparticle coordination polymers (DA-NCPs) have worse toxicity, degradation kinetics, and absorption by BE (2)-M17 DA in vitro with glutamatergic cells and free DA. Direct particle mixing in the ventricle of rats in vivo appears to result in rapid transport within the brain of normal rats, increasing striatal DA levels. More importantly, after 4 days of nasal doses of DA-NCPs equivalent to 200 g of free medication each day, the duration and number of apomorphine-induced rotations were meaningfully fewer than in either vehicle or DA-treated rats used for comparability [172].

Linying Liu and colleagues demonstrated a straightforward technique using a switchable poly (carboxy betaine) gold nanoparticle that delivered genes and chemical medicines programmatically (gene–chem). The particle is replaceable between the delivery route and specific infected cells, allowing for enhanced synergistic neuronal recovery and also computed tomography (CT) imaging. The precise transformation delivery framework will serve as an effective gene–chem co-delivery stage to enable precise brain malady therapy [173].

The effects of polymer-coated silica NPs utilized in laser tissue soldering (LTS) on human brain endothelial cells (ECs) and BBB models were studied by Aniela Bittner et al. In the co-culture scenario with pericytes and ECs, only the cell type directly exposed to NPs demonstrated a time-dependent internalization. There was no evidence of NP transfer between the two cell types. The length and concentration of NP introduction had a moderate effect on cell reasonability. Following NP injection, protein expression of the nuclear factor k-light-chain-enhancer in various endothelial grip particles and activated B cells revealed no irritation or EC enactment. The established endothelial layer reduced the section of judgment tracer particles. The addition of NPs had no effect on junctional proteins, BBB arrangement, or decision making [174].

PLA-coated MSNs were tested for brain targeting with low-density lipoprotein receptor (LDLR) as a ligand. These targeted particles were capable of crossing the BBB and delivering resveratrol to the CNS. PLA was explored in this research as a stimuli-responsive watchman. It was demonstrated that PLA fundamentally impairs medication discharge in PBS, but in the presence of superoxide (ROS), sedate discharge increased due to PLA’s accelerated decomposition [175].

**Figure 9:**
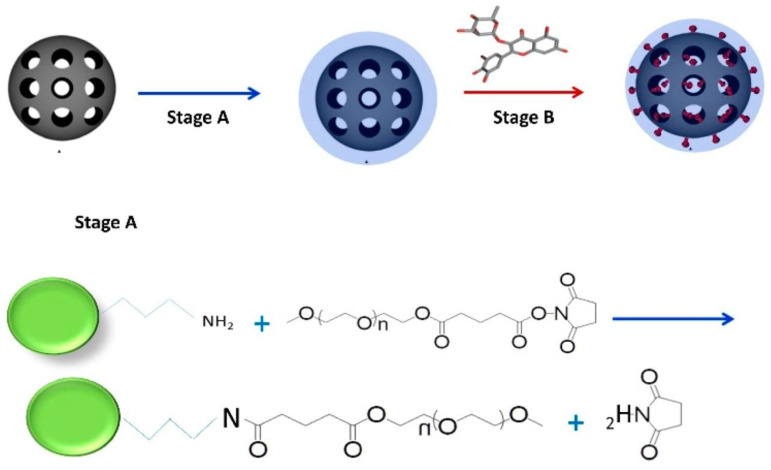
Synthesis of the flavonoid-loaded mesoporous silica nanoparticles (MSNs). Stage A: PEGylation of propylamine MSN; Stage B: Loading of flavonoids, adapted from [176], MDPI, 2019.

PEG-coated MSNs were used to deliver flavonoids (Figure 9) with significant antioxidant effects (quercetin, myricetin, and myricitrin) to increase their water solubility, release characteristics, and stability. Flavonoids with high drug loading (up to 27 weight percent) were found to be effective at inhibiting H_2_O_2_-induced changes in the elasticity and morphology of a model lipid membrane. The findings suggested that flavonoid-loaded MSNs had neuroprotective properties and that atomic force microscopy could be a useful technology for monitoring drug-induced effects at the membrane level, which could be expanded to the cellular level [176].

Cerium oxide nanoparticles appear to mimic oxidoreductase proteins by stimulating the decomposition of reactive oxygen species and natural substrates. Hydrogen peroxides and superoxide radicals, which are damaging particles produced in oxidative stress-related diseases, exhibit this similarity [177].

By affixing 7.8 nm cerium oxide centers to four PEG-grafted copolymers and two poly (sodium acrylate) with various terminal or tying down conclusion bunches, such as phosphonic acids, Victor Baldim et al. created six polymer-coated cerium oxide nanoparticles. The coated nanoparticles’ superoxide dismutase, peroxidase, catalase, and oxidase-like catalytic works were usefully considered. The polymer coatings do not disrupt oxidase-like, superoxide, dismutase-like, or catalase-like, catalytic activities of cerium oxide nanoparticles, but surprisingly boost peroxidase-like catalytic activities. It is also demonstrated that the particles coated with PEG-grafted copolymers work better as oxidoreductase-like proteins than the PAA-coated particles, a finding that supports the use of phosphonic acids as securing bunches at the molecular surface [177].

Xiaoming Chen and colleagues coated PLA microspheres with cationic polymers such as chitosan chloride (CSC), chitosan (CS), and polyethyleneimine (PEI) to form positively charged surfaces. By raising the surface charge of coated microspheres, they appeared to boost antigen adsorption capacity. HBsAg adsorbed on the surface of cationic microspheres improved antigen take-up and increased CD86, MHC I, and MHC II expression, as well as IL-1, IL-6, TNF-, and IL-12 release in macrophages. In antigen-attached cationic microsphere formations, antigens were more likely to relocate independently of lysosomes following phagocytosis. Furthermore, they found that microspheres coated with cationic polymers with somewhat high positive charges and greater antigen adsorption stimulated the Th1 response effectively. In summation, cationic polymer-coated PLA microspheres might serve as a vehicle for recombinant antigen delivery to initiate solid cell and humoral-resistant reactions [178].

Fornaguera C. and coworkers designed novel galantamine-loaded poly (lactic-co-glycolic acid) NPs for the first time by using the nano-emulsification method. They displayed that these NPs have the appropriate features to become advanced drug delivery systems for the therapy of neurodegenerative diseases. Loading proficiency was higher than 90 wt% with a supported medicate release profile. The enzymatic activity of the NPs was kept at 80% after its loading in nanoparticles [179].

Meenakshi Malhotra and colleagues produced nanoparticles for siRNA conveyance in ND using an underutilized peptide-tagged PEG chitosan polymer. They specifically advertised a simple chemo-specific conjugation of monomethoxy PEG at the C2 hydroxyl gather of chitosan polymer, with PEG conjugation to a cell-penetrating peptide. In an in vitro demonstration of an NDD Spinocerebellar ataxia (SCA1) over-expressing ataxin protein, the nanoparticles attempted to transport a useful siRNA against the Ataxin-1 quality. The results show that the SCA1 protein is successfully hidden after 48 h of transfection [159]. The structure and synthesis method of the coated NPs are shown in Table 2. Various polymer-coated nanoparticles for brain-targeted delivery are summarized in Table 3.

## 6. Challenges

Unlike significant achievements in the biomedical application of nanomedicine, there are some problems which have not yet been reported in clinical treatment, and these are limited in experimental data [189]. Importantly, a clear pathway leading to a cure for these diseases remains elusive, and the potential toxicity of NPs and their effect on the human brain are considered some of the main challenges leading to complicated molecular pathogenesis procedures, useless medical protocol, asymptomatic presentation, and the heterogeneous nature of the diseases [189,190]. Many factors including the size, shape, surface potential, surface functionalization, impurity attached during synthesis, and chemical composition have a direct bearing on the toxicity of nanomaterials [191]. In addition, some metal NPs block the protein transformation of heavy-chain ferritin and form complex structures that adversely affect the function of neuroprotection [192].

Accordingly, it seems that there are still unclear and complex challenges for the application of nanomedicine which are necessary to attend to in clinical translation.

## 7. Conclusions and Future Prospective

The cells of the BBB produce prostaglandins, nitric oxide, and cytokines that affect central nervous system (CNS) function, and dysfunction of the BBB causes some neurodegenerative disorders. It is essential to present a sufficient procedure in drug delivery and design suitable nanocarriers which are able to surround drugs to protect them, improve their circulation time, and provide a temporal and spatial controlled release in these areas through some strategies such as the usage of certain ligands on NP surface, exploiting their shape, and nanocarrier-based biocompatible polymers. To date, there has not been a reported case in clinical trials for neurodegenerative disorders, and it seems that it is the best occasion for NP developed in pre-clinical tests to pave the way for treating these diseases. Thus, a NP must be designed that behaves specifically and decreases side effects on other organs and cells. To sum up, the design of compatible, suitable polymer-coated nanoparticles and a combination of favorable therapeutic and imaging agents will be necessary to open the horizon in the treatment of more effective, non-evasive, and brain-directed treatments in neurodegenerative diseases which improve longevity and health.

## Figures and Tables

**Table 1:** The advantages and disadvantages of the common nanoparticles in brain delivery.

Components	Advantages	Disadvantages	Ref.
Cerium oxide	Manageability. Cost-effectiveness. Less time consuming. Required less energy.	Poor ability to overcome the BBB.	[158]
Chitosan	Nontoxic. Biodegradable. Biocompatible polysaccharide. Soluble in dilute acids. Helping in complexing the negatively charged therapeutic molecules simply by ionic interactions. Improving cellular uptake of chitosan-derived nanoparticles (proton sponge effect).	Poor water solubility. Weak encapsulation efficiency of both hydrophilic and hydrophobic drugs.	[159] [160]
Gold	Predominant optical properties fit for detection/imaging. Well-established synthesis methods. The potential capability to cross the BBB. Preventing cognitive deficits, oxidative stress and inflammation in AD rat model.	Relative low cytotoxicity. Finding easy synthesis. Control over size and shape. Improving colloidal stability, and the ability to tune the surface chemistry to achieve easy conjugation.	[78,158]
Silica	Large surface area. High structural stability. Easy surface functionalization. Low cost of production. Excellent biocompatibility. Protracted circulation properties.	Pore size of mesoporous silica. Decrease toxicity. Tuning particle size, pore size, surface properties, and the porous structure. Endowing MSNs with a large surface area for drug adsorption.	[78]

**Table 2:** The structure and synthesis method of the coated NPs.

Component	Synthesis Method	Structure	Ref.
GO/PCL	electrospinning	porous framework	[166]
GO	hummer	nanosheets	[167]
SiO_2_@PSA	ionic gelation method	core-shell	[168]
Ag NPs	precipitation	core-shell	[169]
Polystyrene	---	nanosphere–chelator conjugate	[170]
Chitosan	ionic gelation	nanospheres	[171]
Dopamine	reversible coordination complexation	coordination polymer nanoparticles	[172]
SiO_2_@ PLLA	Stober	core-shell	[174]
Polymer-coated cerium oxide	precipitation−redispersion	stair-like	[177]
Cationic polymer-coated PLA	emulsification and emulsion solvent evaporation	microspheres	[178]

**Table 3:** Various polymer-coated nanoparticles for brain-targeted delivery.

Drug Carriers	Therapeutic Agents	Diseases	Surface Coating	Study Model	Reference
PLA	FITC-dextran	BBB	PS 80	Kunming mice	[180]
PLGA	siRNA	Traumatic brain injury	PS 80	C57BL/6J mice	[181]
PLA-b-PEG	Amphotericin B	Cryptococcal Meningitis	PS 80	BALB/c mice	[182]
Chitosan	Tacrine	Alzheimer’s Disease	PS 80	in vitro release	[183]
liposomes	Galantamine	Alzheimer’s disease and vascular dementia	PEG	rat brains	[184]
Silica-encapsulated liposomes	Arsenic trioxide	Glioma	PAA	rat brains	[160]
MSNs	Resveratrol	Parkinson’s disease	PLA	rat brains	[175]
PLGA	Curcumin	Alzheimer’s disease	Chitosan	Tg2576 mice	[185]
PLGA	Epigallocatechin gallate	Alzheimer’s disease	PEG	APP/PS1 mice	[186]
PLGA	Epigallocatechin gallate	Epilepsy	PEG	KA–C57BL/6 mice	[187]
PLGA	TRH analogs	Epilepsy	Chitosan	rat brains	[188]

## Data Availability

Not applicable.
